# Human metapneumovirus induces more severe disease and stronger innate immune response in BALB/c mice as compared with respiratory syncytial virus

**DOI:** 10.1186/1465-9921-8-6

**Published:** 2007-01-29

**Authors:** Barbara Huck, Dieter Neumann-Haefelin, Annette Schmitt-Graeff, Markus Weckmann, Jörg Mattes, Stephan Ehl, Valeria Falcone

**Affiliations:** 1 Department of Virology, Freiburg University Medical Center, Hermann-Herder-Straße 11, 79104 Freiburg, Germany; 2 Department of General Pathology, Freiburg University Medical Center, Breisacher Straße115a 79002 Freiburg, Germany; 3 Center for Pediatrics and Adolescent Medicine, Freiburg University Medical Center, Mathildenstraße 1, 79106 Freiburg, Germany; 4 Department of Internal Medicine I, University Hospital Heidelberg, Heidelberg, Germany; 5 School of Biomedical Science, University of Newcastle, Newcastle, Australia

## Abstract

**Background:**

Human metapneumovirus (HMPV) and respiratory syncytial virus (RSV) are members of the *Pneumovirinae *subfamily of *Paramyxoviridae *and can cause severe respiratory disease, especially in infants and young children. Some differences in the clinical course of these infections have been described, but there are few comparative data on pathogenesis in humans and animal models. In this study, HMPV and RSV were compared for replication, pathogenesis and immune induction in BALB/c mice infected with equivalent inocula of either virus.

**Methods:**

Viral titers in the lungs and in the nasal turbinates of mice were determined by plaque assay. Histopathological changes in the lungs as well as weight loss and levels of airway obstruction were monitored in the infected mice to record the severity of illness. Inflammatory cells recruited to the lungs were characterized by flow cytometry and by differential staining. In the case of natural killer cells, cytotoxic activity was also measured. Cytokine levels in the BAL were determined by cytometric bead array.

**Results:**

RSV replicated to higher titers than HMPV in the lung and in the upper respiratory tract (URT), and virus elimination from the lungs was more rapid in HMPV-infected mice. Clinical illness as determined by airway obstruction, weight loss, and histopathology was significantly more severe after HMPV infection. A comparison of the cellular immune response revealed similar recruitment of T lymphocytes with a predominance of IFN-γ-producing CD8+ T cells. By contrast, there were obvious differences in the innate immune response. After HMPV infection, more neutrophils could be detected in the airways and there were more activated NK cells than in RSV-infected mice. This correlated with higher levels of IL-6, TNF-α and MCP-1.

**Conclusion:**

This study shows important differences in HMPV and RSV pathogenesis and suggests that the pronounced innate immune response observed after HMPV infection might be instrumental in the severe pathology.

## Background

Human metapneumovirus (HMPV), a newly identified member of the *Pneumovirinae *subfamily of *Paramyxoviridae*, has recently been recognised as a leading cause of acute respiratory tract disease in infants and children worldwide [1]. HMPV also represents a significant etiology of acute respiratory disease in adults, particularly the elderly and those with comorbid conditions such as chronic obstructive pulmonary disease, asthma, cancer [2], or immunodeficiency [3]. The seasonal occurrence as well as the spectrum of clinical illness, ranging from rhinorrhea, cough and wheezing to severe pneumonia, resemble those of the related respiratory syncytial virus (RSV) [4,5], although some differences are apparent. In fact, infants suffering from respiratory tract infections, have lower levels of inflammatory cytokines in nasal secretions, when infected with HMPV than with RSV [6]. On the other hand, HMPV infection is more often associated with a diagnosis of pneumonia than RSV [6-8]. These reports suggest that HMPV biological properties and pathogenesis may differ from those of RSV.

Considerable progress has been made in molecular epidemiology [9] and development of diagnostic assays [10]. Several animal models of HMPV infection, including BALB/c mice, cotton rats, hamsters, ferrets and non human primates, have been established to better understand viral pathogenesis. However, many questions on the implication of viral and host factors in the development of disease still remain open [7,11-16]. In particular, HMPV-related immunopathogenesis and the possibility of viral persistence need further investigation.

RSV infection of BALB/c mice represents a well established experimental model which has successfully been used to study pathogenesis of and immune response to this pneumovirus [17]. Although RSV can directly affect the integrity of the respiratory epithelium, the immune response is the most crucial factor in pathogenesis, and RSV-induced cytokines and chemokines play an important role in regulating illness and inflammation [17]. BALB/c mice have been reported to be semipermissive for HMPV in some studies [11,13,15] but highly permissive in others [7,14,18]. This divergence may be ascribed to differences between HMPV strains, although this has not been reported in hamsters infected with different viral strains [11,12]. The kinetics of HMPV replication in the respiratory tract of mice apparently resembles that of RSV, with peaks of virus replication occurring between 3 and 4 days after infection [11,12]. Only one study using HMPV/CAN98-75 showed biphasic growth kinetics with peak titers occurring at days 7 and 14 post infection [18]. In contrast to RSV, the immune response to HMPV was characterized by a low inflammatory response, minimal innate immunity and limited T cell trafficking to the lung [7]. Although these findings indicate some differences in pathogenesis, comparative data on mice infected with equivalent doses of RSV or HMPV have not been reported.

Here, we directly compare the kinetics of viral replication, pathogenesis, and immune response in the BALB/c mouse model after infection with the same dose of HMPV or RSV using either a low passage-clinical isolate obtained in our laboratory (HMPV/D03-574) and phylogenetically characterized as subtype A2a [19], or the RSV strain A2.

Our results reveal distinct features of host response to HMPV or RSV that correlate with differences in disease severity.

## Methods

### Mice

Eight to 10-week-old, specific-pathogen-free female BALB/c mice were obtained from Charles River (Sulzfeld, Germany). The mice were kept in a venti-rack at the Institute for Medical Microbiology and Hygiene, Freiburg and fed sterilized water and food ad libitum. All experiments were performed in accordance with the local animal care commission.

### Cell lines and viruses

Rhesus monkey kidney cells (LLC-MK2 and Vero) were maintained in D-MEM supplemented with 10% FCS, 1% L-glutamine, and penicillin/streptomycin (complete medium) and Eagles's MEM complete medium, respectively. HMPV was propagated in LLC-MK2 cells cultured in D-MEM w/o serum supplemented with 1% L-glutamine, penicillin/streptomycin antibiotic mix and 5 μg/ml trypsin (Sigma-Aldrich, Munich, Germany) (trypsin medium) whereas RSV was grown on HEp-2 cells cultured in Eagles's MEM complete medium.

For HMPV and RSV titration on Vero cells, Eagle's MEM trypsin medium and Eagle's MEM supplemeted with 5% FCS were used, respectively.

The HMPV strain D03-574 (subgroup A2a) was isolated from an infant with bronchiolitis in our laboratory, propagated 4 times on LLC-MK2 cells and used to prepare a virus stock. Virus was harvested 3 days p.i., snap-frozen, and kept in liquid nitrogen. The infectious virus titer of the stock was 2,5 × 10^6 ^plaque froming units/ml (PFU/ml). The RSV A2 strain is a common laboratory strain and was originally obtained from Peter Openshaw (Imperial College, London, GB), grown on HEp-2 cells over 4 passages, and kept in liquid nitrogen. The infectious virus titer of the stock on Vero cells was 3,3 × 10^7 ^PFU/ml.

### Infection, organ collection, and virus titration

BALB/c mice were lightly anesthetized by intraperitoneal injection of ketamine (2 mg/mouse) and xylazine (0.15 mg/mouse), and infected intranasally (i.n.) with 2 × 10^5 ^PFU HMPV strain D03-574 or 2 × 10^5^(when indicated 10^6^) PFU RSV strain A2 in 80 μl serum-free (SF) Eagle's MEM.

At the indicated time points, anesthetized mice were exsanguinated, and lungs and nasal turbinates were harvested separately for virus quantification by plaque assay. Lungs were homogenized using a Teflon pestle in a volume of 1.5 ml of SF culture medium, whereas nasal turbinates were homogenized in 3 ml by grinding with sterile sand. Total homogenates were quickly spun down, and supernatants were frozen in liquid nitrogen until use. To determine HMPV titers, 150 μl of 10-fold serial dilutions of the clarified homogenates were added in duplicate to confluent Vero cell monolayers in a 24-well plate and cultured for 5 days under 0.8% methylcellulose, followed by fixation with 80% methanol. HMPV plaques were visualized by incubation with anti-HMPV rabbit serum (kindly provided by U. Buchholz, NIH, Bethesda, MD, USA) followed by incubation with anti-rabbit IgG coupled to biotin (Perbio Science Deutschland, Bonn, Germany) and with streptavidin coupled to horseradish peroxidase (SA-HRP; BD PharMingen, San Diego, CA). Finally, the plaques were enumerated after addition of 3',3'-diaminobenzidine substrate (DAB; Merck, Darmstadt, Germany). Similarly, RSV titers were determined on Vero cells and detected using a biotin-labelled anti-RSV antibody (Biogenesis, Berlin, Germany) followed by incubation with SA-HRP and DAB substrate as previously described [20].

### Determination of pulmonary function by whole-body plethysmography

Whole-body plethysmography (Buxco Electronics Inc. Troy, NY) was used, to monitor the respiratory dynamics of mice in a quantitative manner. Penh is a dimensionless value that represents a function of the ratio of peak expiratory flow to peak inspiratory flow and a function of the timing of expiration, and it correlates with pulmonary airflow resistance. Penh has previously been validated in animal models of airway hyperresponsiveness (AHR) [21,22] and infection-associated airway obstruction (AO) [23]. Baseline airway resistance (with or without infection) is described as AO and the transient airway resistance in response to methacholine as AHR. Before exposure to methacholine, mice were allowed to acclimate to the plethysmograph chamber, and then baseline readings were recorded to determine AO. Mice were exposed to increasing doses of aerosolized methacholine/mL (Sigma-Aldrich). Plethysmograph readings were recorded again to determine AHR. Groups of infected and control mice were always evaluated in parallel.

### Analysis of cellular lung infiltrates

Pulmonary inflammatory cells were obtained by bronchoalveolar lavage (BAL) as previously described [20] and used for NK assay or antibody staining without further manipulations. For microscopic differentiation of BAL macrophages, neutrophils, and lymphocytes, 10^4 ^BAL cells were used for cytospin preparation on glass slides utilizing a Shandon Cytospin centrifuge (Thermo Electron, Waltham, MA). After air-drying, 2 ml Wright's staining solution (FLUKA, Buchs, Austria) was added for 6 minutes, followed by water for 6 minutes. After washing and air-drying, a differential cell count was performed with 200 cells.

### Natural killer (NK)cell assay

The NK cell assay was performed under "mini-killer" conditions as previously described [24]. Briefly, effector BAL cells were plated in two-fold dilutions starting with 5 × 10^4 ^cells per well in a volume of 50 μl in a 96-well V-bottom plate (Greiner Labortechnik, Solingen, Germany). YAC-1, labelled with (^51^Cr), were used as target cells, and 2 × 10^3 ^cells were added in a volume of 50 μl per well for an initial effector:target ratio of 25.

### Flow cytometry

Single-cell suspensions of BAL cells (10^5^) were surface-stained for 30 min at 4°C with the following antibody combinations: (i) anti-CD8-FITC (Ly-2; clone 53-6.7), anti-CD4-PE (L3T4; clone RM 4–5) and anti-CD3-APC (CD3 ε chain; clone 145-2C11); (ii) anti-CD3-APC and anti-DX5-bio (CD49b/Pan-NK), followed by incubation with SA-Cy (all from BD PharMingen, San Diego, CA).

To detect intracellular cytokines, cells (1–2 × 10^5^) were incubated with 50 ng/ml phorbol myristate acetate (PMA) (Sigma-Aldrich), 500 ng of ionomycin (Calbiochem, San Diego, CA) and 1 μl/ml monensin (Golgistop, BD PharMingen) for 5 h at 37°C. Cells were harvested, washed and surface-stained with anti-CD3-APC and anti-CD8-FITC and then subjected to intracellular cytokine staining using the cytofix/cytoperm kit according to the manufacturer's instruction (BD PharMingen). Cells were stained with anti-IFN-γ-PE or with isotype-control antibody (BD PharMingen). After 30 min, cells were washed and analyzed on a fluorescence-activated cell sorter (FACScan and Cellquest Software, Becton Dickinson, Heidelberg, Germany), collecting data on at least 10,000 lymphocytes. Calculations of percentages were based on live cells as determined by FSC/SSC analysis.

### Analysis of secreted cytokines by cytometric bead array

BAL supernatants obtained by centrifugation of BAL cells for 10 min at 1600 rpm were harvested and stored at -70°C until cytokine testing was performed. IL-6, IL-10, IL-12, MCP-1, TNF-α, and IFN-γ were detected simultaneously using the Cytometric Bead Array (CBA) Mouse Inflammation Kit (BD PharMingen). Briefly, 50 μl of each sample was mixed with 50 μl of mixed capture beads and 50 μl of the mouse Th1/Th2 PE detection reagent consisting of PE-conjugated anti-mouse IL-6, IL-10, IL-12, MCP-1, TNF-α, and IFN-γ. The samples were incubated at room temperature for 3 h in the dark. After incubation with the PE detection reagent, the samples were washed once and resuspended in 300 μl of wash buffer before acquisition on a FACScan cytometer. Data were analyzed using CBA software (BD PharMingen). Standard curves were generated for each cytokine using the mixed cytokine standard provided by the kit. The concentration for each cytokine in cell supernatants was determined by interpolation from the corresponding standard curve. The range of detection was 20–5000 pg/ml for each cytokine measured by CBA.

### Histopathology and immunohistochemistry

For histological examination, lung specimens from RSV- and HMPV-infected mice and normal control mice were collected and fixed in 4% buffered formalin. Paraffin-embedded tissue blocks were cut at 4 μm. Deparaffinized sections were evaluated following hematoxylin & eosin (H&E) staining or by immunolabelling using anti-HMPV rabbit serum. Briefly, immunohistochemistry was performed after antigen retrieval and incubation for 20 min with 10% blocking serum (Biotrend, Cologne, Germany). Sections were stained semiautomatically (Autostainer instrument, Dako, Hamburg, Germany) with the primary antibody and a biotinylated detection antibody. Antibody binding was detected by the labelled streptavidin-biotin (LSAB) method [25] (ChemMate K5005 Alkaline Phosphatase/Red detection kit, Dako). Nuclei were counterstained with Mayer's hemalaun solution.

### Statistical analysis

All data are expressed as mean +/- standard deviation. Student's unpaired *t*-test was used to compare HMPV-infected and RSV-infected animals at the same time point (significance level set at *P *< 0.05).

## Results

### HMPV and RSV replication in the respiratory tract of BALB/c mice

**Figure 1:**
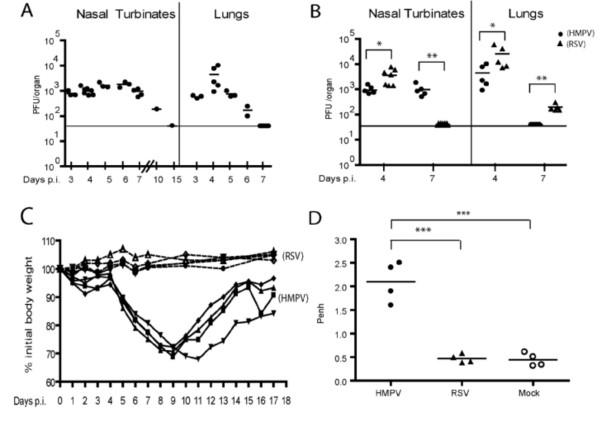
Course of HMPV and RSV infection in the BALB/c mice. (A) Kinetics of HMPV replication in the respiratory tract. Animals were infected i.n. with 2 × 10^5 ^PFU of HMPV and viral titers were determined in nasal turbinates and lungs at the indicated time points. (B) Comparison of HMPV and RSV titers measured on day 4 and 7 p.i. in nasal turbinates and lungs of mice infected with equivalent doses (2 × 10^5 ^PFU) of either virus preparation. * P < 0.05, ** P < 0.01, (*n *= 5–7). (C) Weight curves of HMPV- or RSV-infected BALB/c mice. Four animals per group were infected i.n. with 2 × 10^5 ^PFU of either HMPV (—) or RSV (----) and weight was recorded daily. The experiment was repeated three times with similar results. (D) Airway obstruction following HMPV, RSV or mock infection of BALB/c mice. Airway function was determined by measuring enhanced pause (Penh) via whole-body plethysmography on day 7 p.i.. *** P < 0.001, (*n *= 4).

To assess whether our HMPV isolate is able to replicate in the respiratory tract of BALB/c mice, animals were infected with 2 × 10^5 ^PFU of HMPV and the kinetics of viral load was determined in the nasal turbinates (upper respiratory tract; URT) and in the lungs (lower respiratory tract; LRT). A virus peak was observed between day 4 and day 6. The virus was eliminated by day 7 from the lungs and between day 10 and day 15 from the URT (Fig 1A). There was no evidence for long-term viral persistence as shown by sensitive real time PCR at day 120 p.i. (data not shown).

To compare HMPV and RSV replication in both compartments, BALB/c mice were infected i.n. with 2 × 10^5 ^PFU of either virus preparation, and virus titers were determined in the URT and in the LRT. On day 4 p.i., titers were higher for RSV than for HMPV (Fig 1B). Consistent with this, HMPV was eliminated from the lungs by day 7 p.i., while low titers of RSV were still present. By contrast, virus elimination from the nasal turbinates was more efficient for RSV whereas significant titers of HMPV were still measured on day 7 p.i.

### Clinical manifestations of HMPV and RSV infection

Mice infected with 2 × 10^5 ^PFU of HMPV or RSV were observed daily for development of clinical symptoms. Unexpectedly and in contrast to the viral replication kinetics, HMPV but not RSV infection was associated with severe weight loss (Fig. 1C). At the peak of weight loss, mice had ruffled fur and showed heavy breathing as well as reduced activity, but all mice finally recovered.

Penh values, representing airway obstruction (AO), were measured on day 7 p.i., in both groups of mice. In accordance with the weight loss and other clinical signs of disease, AO was significantly higher in HMPV- than in RSV-infected mice or uninfected controls. This confirmed the inverse relationship between viral replication and the pathological changes (Fig. 1D).

Due to high baseline Penh values in HMPV-infected mice, AHR measurement after methacoline treatment did not yield significant results (data not shown).

### Histopathological changes in lungs

**Figure 2:**
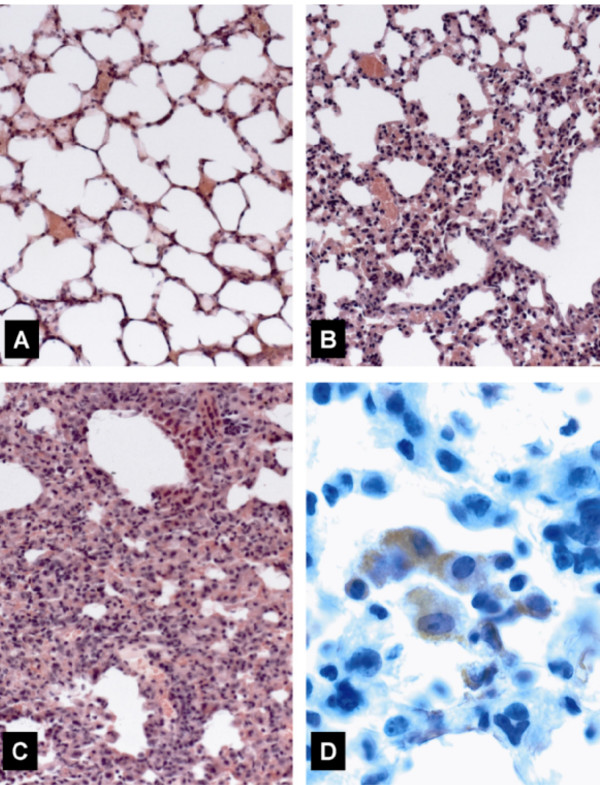
Immunohistochemistry of lungs on day 7 after infection with HMPV or RSV. (A) Normal lung tissue from an uninfected animal. (B) Pulmonary section from an RSV-infected mouse showing mild bronchopneumonia with scattered macrophages and neutrophils in alveolar spaces. (C) Severe bronchopneumonia in a mouse inoculated with HMPV. Bronchioli and adjacent alveoli are densely infiltrated by macrophages and neutrophils admixed with fibrin. (D) Immunohistochemical staining for HMPV. Groups of intraalveolar macrophages and pneumocytes expressing HMPV antigens. (A to D): hematoxylin-eosin staining, original magnification × 20; D: immunostaining with anti-HMPV serum, (× 63). Representative sections from groups of 4 mice are shown.

Hematoxylin-eosin-stained lung sections obtained from RSV-infected mice on day 7 p.i. revealed rare foci of mild inflammatory infiltration of bronchioles and adjacent alveoli, while no histopathology was found in uninfected animals (Fig. 2A and 2B). By contrast, bronchioli and pulmonary parenchyma of HMPV inoculated mice showed severe bronchopneumonia. Bronchioli and alveolar spaces were densely packed with neutrophils, lymphocytes, macrophages, desquamated pneumocytes, and fibrin (Fig. 2C). Immunolabelling revealed groups of intraalveolar macrophages and pneumocytes expressing HMPV antigens (Fig. 2D).

### Characterization of cellular infiltrates after HMPV and RSV infection

**Figure 3:**
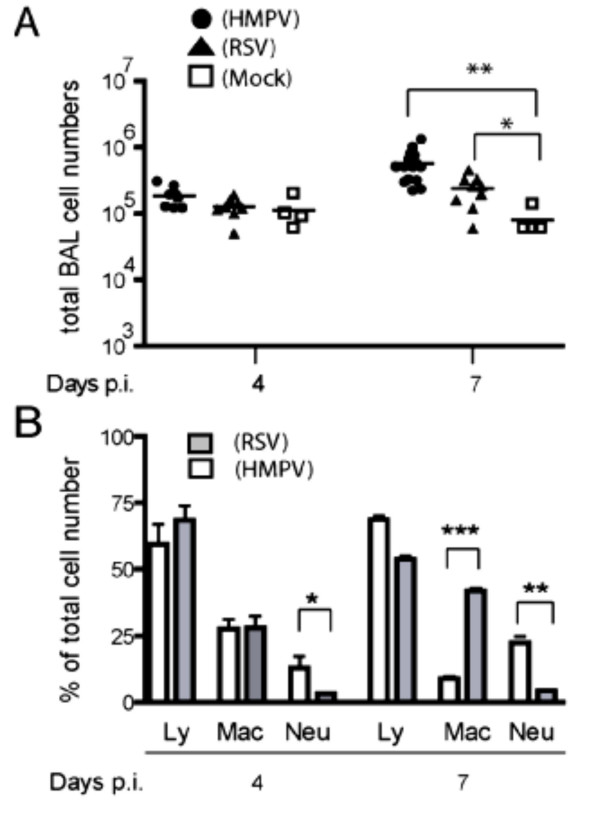
Cellular infiltration of the lungs after HMPV or RSV infection. (A) Total BAL cells in HMPV-, RSV-, and mock-infected mice. (B) Differential count of BAL cells from HMPV- and RSV-infected mice after Giemsa staining. Ly, lymphocytes; Mac, macrophages; Neu, neutrophils. * P < 0.05, ** P < 0.01, ***P < 0.001 (*n *= 4–10)

**Figure 4:**
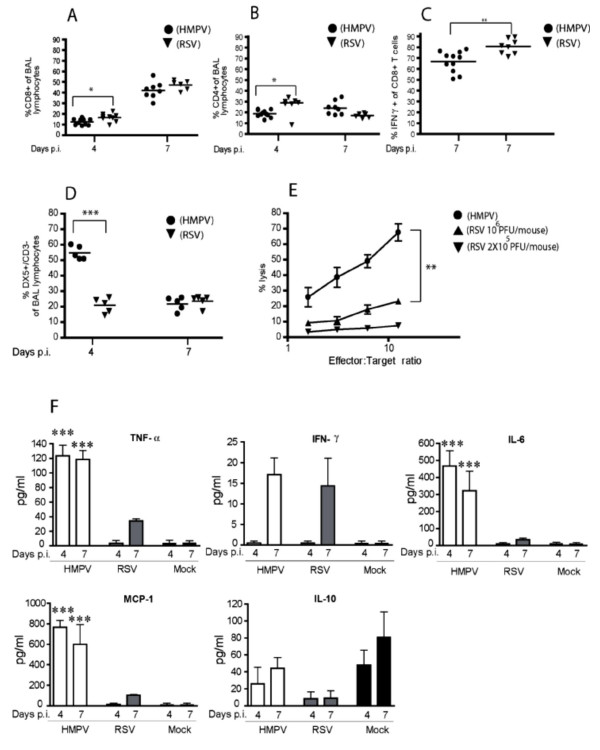
Characterization and functional analysis of BAL cells after HMPV or RSV infection. Percentage of (A) CD8+, (B) CD4+, and (C) IFN-γ-producing CD8+ cells of total BAL lymphocytes as determined by FACS analysis of BAL cells from HMPV- or RSV-infected BALB/c mice. * P < 0.05, ** P < 0.01 (n = 6–11). (D) Percentage of NK cells (DX5+/CD3-) of total BAL lymphocytes in the BAL of HMPV- or RSV-infected BALB/C mice (2 × 105 PFU/mouse), and (E) NK cell-mediated cytotoxicity in mice infected with 2 × 105 PFU of HMPV or 2 × 105 and 106 PFU of RSV, respectively. The experiment was repeated three times with similar results. ***P < 0.001 (n = 5). (F) Cytokines in the BAL of HMPV- or RSV-infected mice. BALB/c mice were infected with 2 × 105 PFU of HMPV or RSV. BAL fluid was collected at different time points after infection and TNF-α, IFN-γ, IL-6, MCP-1, and IL-10 were measured by cytometric bead array. Values represent mean +/- SEM. ***P < 0.001 (n = 4).

To further understand the inverse correlation between viral replication and clinical disease during infection with HMPV versus RSV, we analyzed the inflammatory cells recruited to the lungs on day 4 and 7 p.i.. Day 4 represents the time of maximal virus replication and accumulation of innate immune cells including NK cells, while day 7 is the time point of maximal T cell recruitment and activity in RSV-infected mice. The number of inflammatory cells eluted from the lung airways by BAL was similar on day 4 p.i. in the HMPV- and in the RSV-infected mice. On day 7 p.i., an increase of total BAL cells was observed in both groups, but HMPV-infected mice had significantly higher numbers of BAL cells (Fig. 3A). Microscopic differentiation of BAL cells revealed that lymphocytes represent the main population at both time points, with little differences among the two groups (Fig. 3B). By contrast, the percentage and absolute numbers of neutrophils was significantly higher in the HMPV- than in the RSV-infected mice at both time points, while macrophages were more prominent at day 7 in RSV-infected mice. Analysis by flow cytometry showed that 7 days after infection CD8+ T cells were the predominant population of lymphocytes in both infection models (Fig. 4A). The percentage of CD4+ T cells was slightly higher after RSV infection on day 4, but no significant differences were observed on day 7 (Fig. 4B). Functional activity of CD8+ T cells at day 7 was evaluated by intracellular IFN-γ staining after in vitro stimulation with PMA-ionomycin (Fig. 4C). A high proportion of CD8+ T cells produced IFN-γ after both infections, but there was a trend towards higher levels of IFN-γ-producing cells after RSV infection. Overall, there were little differences in T cell recruitment to the respiratory tract during the two infections arguing against a role for T cells in the differences between HMPV and RSV-induced pathology.

### High numbers of NK cells recruited to the lungs after HMPV but not RSV infection

NK cell accumulation in the BAL of HMPV- and RSV-infected animals was determined by flow cytometry. The percentage of NK cells (DX5+/CD3- cells) recruited to the lungs of HMPV-infected mice at day 4 p.i. was significantly higher than in RSV-infected mice. The DX5+/CD3- population declined thereafter and at day 7 comparable numbers of NK cells were present following infection with both viruses (Fig. 4D). At day 4 p.i., NK cell cytotoxicity was assessed by direct ex vivo cytotoxicity of BAL cells against YAC-1 target cells. NK cell activity was significantly higher after infection with HMPV than after infection with the same dose of RSV (Fig. 4E). NK cell recruitment and activity was increased in mice infected with a 5-fold higher RSV inoculum (10^6 ^PFU/mouse) but was still less than that observed in mice infected with the lower HMPV dose (Fig. 4E and data not shown). Thus, HMPV appears to be a more potent inducer of NK cell activity than RSV.

### Different pulmonary cytokine responses after HMPV and RSV infection

To further characterize the factors that regulate HMPV pathogenesis in the mouse model, we analyzed the production of cytokines and chemokines by BAL cells. For this purpose, cell-free BAL fluids obtained at day 4 and at day 7 p.i. were analyzed by cytometric bead array for the presence of TNF-α, IFN-γ, IL-6, IL-10, and MCP-1. Consistent with the levels of cellular infiltration observed, HMPV-infected mice produced significantly higher levels of TNF-α, IL-6, and MCP-1 than RSV-infected mice at both, day 4 and day 7 p.i.(Fig. 4F), but similar levels of IFN-γ were measured at day 7 p.i.. Interestingly, infection with RSV, but not with HMPV, seemed to downregulate IL-10, which is produced to discrete levels after mock infection. Overall significant differences were observed in the amount of inflammatory mediators after the two infections.

## Discussion

This study shows that pulmonary infection of mice with equivalent doses of RSV and HMPV leads to different clinical outcomes. Although RSV replicated to higher titers, HMPV caused more severe disease associated with higher levels of cytokines and a much stronger NK cell response. These findings indicate important differences in the pathogenesis of respiratory disease induced by these two related paramyxoviruses.

HMPV reached lower titers than RSV, both in the URT and LRT, with a single peak observed on day 4 in both infection models (Fig. 1A and 1B). An early report had suggested that HMPV replicates in lung tissue with biphasic kinetics reaching peak titers 7 and 14 days p.i[18]. By contrast, more recent results in the mouse and in the cotton rat model [13,14,16], showed uniphasic growth kinetics, more consistent with our results and similar to what can be observed after RSV infection [17]. Lower HMPV peak titers might reflect different susceptibility of airway epithelial cells to viral infections or viral spread; in addition, they may indicate differences in the early (innate) immune response. Indeed, we found evidence that the NK cell response is much stronger in HMPV- than RSV-infected mice.

Despite poor replication, HMPV induced considerable airway obstruction, weight loss, and histopathology, while only minimal changes occurred in RSV-infected mice.

A closer look at the inflammatory cell infiltrates revealed significant differences in two components of the innate immune response. In particular, HMPV induced a more prominent recruitment of neutrophils and NK cells to the BAL when compared to RSV (Fig. 3B and 4D). These findings support the concept that HMPV may elicit a more pronounced innate immune response that, on the one hand, is beneficial for virus control but, on the other hand, may cause more extensive immunopathology. Previous data have shown that RSV infection in the BALB/c mouse leads to recruitment of neutrophils and NK cells to the lungs, with a peak observed on day 4 p.i. [26]. We found that, even after infection with a 5-fold higher inoculum, RSV was not able to recruit and activate NK cells to the same extent as HMPV (Fig. 4D and 4E). However, the potential role of this cell subset for HMPV pathogenesis has to be clarified by future approaches, such as in vivo depletion of NK cells.

It has been suggested that the RSV G and/or SH protein inhibit trafficking of NK cells to the lungs, since the absence of the corresponding genes markedly increases the number of NK cells in BAL [27]. The mechanism of this inhibition is still unknown, but it might be an effect of these proteins on the profile of chemokines produced [28]. Therefore, the structural differences between the G and the SH proteins of HMPV and RSV might be instrumental in recruiting NK cells to high levels. However, this possibility needs to be further evaluated.

Higher levels of the inflammatory cytokines IL-6, TNF-α and of the C-C chemokine MCP-1 were observed in HMPV-compared to RSV-infected mice. This is in contrast to previous findings showing that HMPV poorly activates inflammatory cytokines such as IL-1, IL-6 and TNF-α [29]. The discrepancy can possibly be assigned to the different properties of the isolates used for the infection studies (low passage clinical isolate in our study *versus *extensively cell-passaged isolate [18]). In fact, pathogen-specific factors may be altered after extensive cell culture passages thus influencing the replication pattern of and the response to a given pathogen. For instance, it has been shown that a non-pathogenic variant of pneumonia virus of mice, another member of the subfamily *Pneumovirinae*, was generated during in vitro passages [30].

It has been reported that RSV induces significant changes in the mouse model only if given at high dose [17,31]; therefore, the low levels of cytokines observed in our study after RSV infection could also be a consequence of the different viral dose used *i.e*. 2 × 10^5 ^PFU/animal in our study *versus *10^7 ^PFU/animal in previous studies [16,23]. Hence, it appears that the virus load required to trigger an inflammatory response (cytokine production as well as recruitment of inflammatory cells) is significantly lower in the HMPV than in the RSV infection process.

In contrast to the NK cell response, the T cell recruitment to the lung airways showed no major differences between the two viral infections. In the absence of defined CTL epitopes, the functional analysis of T cells was restricted to IFN-γ production following non-specific PMA/Ionomycin stimulation and was found to be slightly lower after HMPV infection than after RSV infection. However, in the absence of data on virus-specific T cell response, these findings should not be over-interpreted.

In pediatric patients, HMPV has been reported to cause a disease pattern similar to that of RSV with signs and symptoms ranging from severe cough to bronchiolitis and pneumonia [5,32]. In some studies but not in others, HMPV infection has been associated more frequently than RSV with acute asthma exacerbations in children [8,33,34] and adults [35] and with more severe lower respiratory tract involvement leading to pneumonia [4,8,32]. In infants, HMPV has been reported to promote a weak inflammatory response, with low levels of cytokines and chemokines in respiratory secretions [6]. By contrast, in a recent study, restimulation by HMPV of human PBMC from previously exposed adults resulted in markedly more robust IL-6 and significantly weaker IFN-γ response than did restimulation by RSV [36]. Taken together, these studies indicate that, as in our mouse model, HMPV-induced pathogenesis may differ significantly from that related to RSV.

## Conclusion

In the present work, direct comparison of HMPV and RSV infection in the mouse model using equivalent inocula under identical conditions has indicated important differences in the response to infection with two distinct viruses of the *Pneumovirinae *subfamily, both responsible for a significant burden of disease in infants and young children. The data suggest that the pronounced NK cell recruitment and activation together with the production of inflammatory cytokines and chemokines might play a crucial role in HMPV-related immunopathogenesis.

## Competing interests

The author(s) declare that they have no competing interests.

## Authors' contributions

BH carried out the titration experiments and the flow cytometry analysis and participated in designing the study and in drafting the manuscript. DNH coordinated the study and participated in writing the manuscript. ASG carried out the histological analysis of mouse lungs and helped to draft the manuscript. MW and JM established and carried out the whole-body plethysmography experiments. SE participated in the design and coordination of the study and in writing the manuscript. VF conceived the study, participated in its design, carried out titration and flow cytometry experiments and wrote the manuscript.
